# Role of Cholinergic Signaling in Alzheimer’s Disease

**DOI:** 10.3390/molecules27061816

**Published:** 2022-03-10

**Authors:** Zhi-Ru Chen, Jia-Bao Huang, Shu-Long Yang, Fen-Fang Hong

**Affiliations:** 1 Experimental Center of Pathogen Biology, Nanchang University, Nanchang 330031, China; zhiru.chen@se19.qmul.ac.uk (Z.-R.C.); j.huang@se19.qmul.ac.uk (J.-B.H.); 2 Queen Mary School, Nanchang University, Nanchang 330006, China; 3 Department of Physiology, Fuzhou Medical College, Nanchang University, Fuzhou 344099, China; 4 Department of Physiology, College of Medicine, Nanchang University, Nanchang 330006, China

**Keywords:** Alzheimer’s disease, cholinergic signaling, neurodegenerative disease, drugs and treatment of Alzheimer’s disease

## Abstract

Acetylcholine, a neurotransmitter secreted by cholinergic neurons, is involved in signal transduction related to memory and learning ability. Alzheimer’s disease (AD), a progressive and commonly diagnosed neurodegenerative disease, is characterized by memory and cognitive decline and behavioral disorders. The pathogenesis of AD is complex and remains unclear, being affected by various factors. The cholinergic hypothesis is the earliest theory about the pathogenesis of AD. Cholinergic atrophy and cognitive decline are accelerated in age-related neurodegenerative diseases such as AD. In addition, abnormal central cholinergic changes can also induce abnormal phosphorylation of ttau protein, nerve cell inflammation, cell apoptosis, and other pathological phenomena, but the exact mechanism of action is still unclear. Due to the complex and unclear pathogenesis, effective methods to prevent and treat AD are unavailable, and research to explore novel therapeutic drugs is various and active in the world. This review summaries the role of cholinergic signaling and the correlation between the cholinergic signaling pathway with other risk factors in AD and provides the latest research about the efficient therapeutic drugs and treatment of AD.

## 1. Introduction

Alzheimer’s disease, the most common type of dementia, is a progressive and commonly diagnosed neurodegenerative disease [1] with a very high mortality rate, which is closely related to genetic factors and age. AD is a major cause of dementia which is a common disease among the elderly, starting with an irreversible decline in episodic memory and then a more general decline in overall cognitive ability [2,3]. AD is characterized by disorientation and gradual deterioration of memory and intelligence [4,5]. About 12% of people over 65 are affected. WHO estimates that more than 48.6 million people worldwide are living with the disease [6]. The increasing number of aging populations globally increases the incidence of chronic degenerative diseases, central nervous system damage and dementia such as AD [7].

AD occurs in the form of autosomal dominant or sporadic genetic diseases [8]. The pathological features of AD are mainly characterized by the deposition of a great deal of senile plaques (SP) in the cerebral cortex and hippocampus, loss of functioning neurons and synapses in certain brain regions, and acetylcholine deficiency. The main clinical manifestations are progressive memory loss and cognitive decline, accompanied by behavioral disorders and symptoms of various neurological diseases. At present, there are many hypotheses for AD, such as the cholinergic hypothesis, Aβ deposition hypothesis, au protein hypothesis and neuroinflammation hypothesis, but the exact pathogenesis of AD still remains unclear. AD accounts for 60–70% of the 50 million cases of dementia worldwide, and is characterized by cognitive decline and has long been associated with dysregulation of the cholinergic system [9,10]. The cholinergic hypothesis is the earliest theory about the pathogenesis of AD, which is significant in AD pathogenesis. Acetylcholine (ACh) is an important excitatory neurotransmitter involved in learning, memory and other higher behaviors. The level of ACh can be affected by the central cholinergic nervous system through regulating the synthesis and release of Ach [11]. Basic forebrain cholinergic neurons (BFCNs) are of significance in learning, memory, and cognitive function [12]. Their survival and differentiation depend on nerve growth factor (NGF) and they dominate cortical and hippocampal areas involved in memory and learning [13]. The hippocampus is a brain region rich in nicotinic acetylcholine receptors (NACHR) [14]. Patients with AD always have degeneration of deep and early basal forebrain cholinergic neurons (BFCN). The degree of dementia is related to synaptic loss between the basal forebrain and the target tissues of the hippocampus and cortex, which is considered to be the main cause of memory decline [15]. According to clinical data in recent years, the brains of patients with AD show severe neurodegeneration and reduction of cholinergic neurons and a severe deficiency of Ach; acetylcholine transferase activity is significantly decreased, which further illustrates the damage to the cholinergic system in patients with AD after serious injury [16]. Thus, restoring the cholinergic system after injury is currently a clinical therapy for the treatment of AD. Acetylcholinesterase (AChE) directly binds to presenilin-1 (PS-1), an important enzyme in the process of Aβ production, and enhances its expression, thereby increasing the level of Aβ, which accelerates cognitive dysfunction [17,18]. In addition, abnormal central cholinergic changes can also induce abnormal phosphorylation of tau protein, nerve cell inflammation, cell apoptosis, neurotransmitter and neurohormone system imbalance and other pathological phenomena, but the mechanism of action is still unclear. AD not only causes serious and irreversible damage to patients’ life and mental quality, but also increases the economic and medical burden on their families and society. The search for a drug to treat AD has become extremely urgent.

This review mainly describes the physiological function of the cholinergic system and how its abnormality accelerates the course of AD, and summarizes the current common drugs related to treatment of AD as well as new drugs and therapies that are still in clinical trials and have good therapeutic potential.

## 2. Physiological Function of Cholinergic System

**Figure 1:**
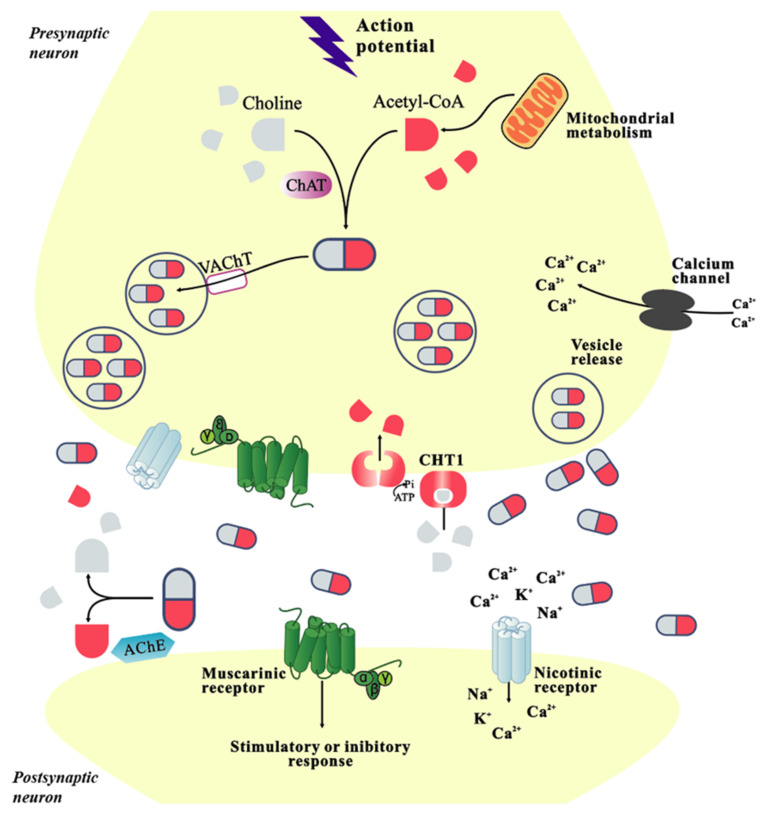
Schematic diagram of choline neurotransmission. ACh is synthesized by choline acetylcholine transferase (ChAT) from the cytoplasm of cholinergic presynaptic neurons by acetyl-coA and choline acetylcholine transferase. It is then transferred to synaptic vesicular follicles via vesicular acetylcholine transporter (VAChT). Depolarization of presynaptic neurons promotes extracellular secretion of ACh, which can then bind to nicotine or muscarinic receptors, leading to stimulatory or inhibitory responses. AChE rapidly hydrolyzes acetylcholine at the synaptic cleft, releasing acetate and choline, which are reabsorbed by the high-affinity choline transporter (CHT1) into presynaptic cholinergic neurons.

Cholinergic neurons have a wide distribution in the human brain and play a role in cognition. Cholinergic signal transduction related to memory and learning depends on Ach [19]. Ach is not only an effective modulator, but also a necessary condition for the full expression of sensation-induced neurovascular coupling responses [20]. It is synthesized by a key enzyme, the choline acetyl transferase. Substrates including choline, acetyl-CoA and ATP, are required for its catalytic activity (Figure 1) [21]. ACh signaling is critical for cognition and suppression of inflammation. Cholinergic signaling is regulated at multiple levels and bidirectionally by proteins and non-coding small RNA (CholinomiRs) to maintain homeostasis. The cognitive and inflammatory aspects of cholinergic signaling are mediated by CholinomiRs by targeting major cholinergic transcripts including acetyl cholinergic hydrolase AChE [22]. In neuronal signaling, human acetylcholinesterase (AChE) is an important enzyme, responsible for the degradation of ACh, which in turn blocks postsynaptic signal transmission (Figure 1) [23]. Cholinergic neurotransmission plays a key role in neuronal plasticity and cell survival in the central nervous system. Two types of AChRs, muscarinic AChRs (mAChRs) and nicotinic AChRs (nAChRs) (Figure 1), trigger intracellular signaling via G-protein activity and ion influx, respectively [24]. In human brain, ACh is absorbed by a special transport system that has different pharmacological properties from the known organic cation transporters [25]. The cholinergic system has a crucial role in regulating neurogenesis, neuronal differentiation, synaptic plasticity and neuroprotection in the central nervous system [26]. At physiological concentrations, Aβ1-42 and Aβ1-16 induce dephosphorylation of synapsin 1, leading to calcineurin activation, which increases the size of the synaptic vesicles (SV) circulating pool. This process enhances neuroplasticity and memory formation by increasing presynaptic release of neurotransmitters [27]. Cholinergic signaling is essential for cognitive function. Basal forebrain is the primary output site of cholinergic energy in the central nervous system [28]. The central cholinergic circuit is involved in maintaining normal cognitive function and regulating signal transduction throughout the cerebral cortex. However, degeneration of central cholinergic neurons may occur, given the anti-inflammatory and antioxidant effects of neuronal glial cholinergic signaling, leading to impaired learning, memory, sleep regulation and attention [29].

Basic forebrain cholinergic neurons (BFCNs) have an important role in learning, memory, and cognitive function [12]. Their survival and differentiation depend on nerve growth factor (NGF) and dominate cortical and hippocampal areas involved in memory and learning processes [13]. The hippocampus is a brain region rich in nicotinic acetylcholine receptors (NACHR), particularly the α7 subtype [14]. Memory formation is associated with downregulation of miR-1, miR-10, and miR-26 expression in the prefrontal cortex (PFC) and hippocampus. Memory formation increases BDNF and p-CREB/CREB in PFC, the hippocampus and amygdala [30]. Dysfunction or degeneration of BFCNs can lead to cholinergic atrophy and cognitive decline, and affect normal function of the hippocampus; it may be associated with the development of neuroinflammatory components of diseases such as AD [31]. The nucleus basalis of Meynert (NBM) is a key brain region that is mainly cholinergic. It may have a key function in coordinating cortical activity throughout the learning process, as novelty, learning and reward expectation are encoded by different NBM signals. Its cellular degradation is associated with the progression of AD and Parkinson [32].

Lipids are not only the main component of cell membranes, participating in metabolism, but also serve as intracellular signal molecules and specific membrane receptors, controlling cell proliferation and growth, and delivering neuroprotection. The specific distribution of different lipids supports their participation in structural and metabolic functions and intracellular effectors, specific ligands and precursors. In addition, localized measurements of the central nervous system allow us to analyze lipid distribution to determine physiological conditions in AD. In the past few decades, the development of mass spectrometry imaging has contributed to our understanding of lipids’ role in pathological conditions in AD. New analytical techniques allow researchers to obtain a wide range of information about the spatial distribution and abundance of different lipid molecules, which is critical to understanding brain function. For example, different kinds of phosphatidylcholine, phosphatidic acid, phosphoinositol and phosphatidylserine are differently distributed in different locations of gray matter and white matter [33]. Transverse membrane asymmetric lipid distribution induces special membrane biophysical properties. AD is related to perturbations of different membrane properties, and nicotinic acetylcholine receptors have been widely correlated with membrane properties [34].

## 3. AD Relation to the Cholinergic System

AD can cause a variety of cellular changes, including impaired cholinergic system, Aβ accumulation, tau hyperphosphorylation, dyshomeostasis of metal, neuroinflammation and many other pathways which are related to the pathogenesis of the disease [35,36]. The link between histopathological markers of AD, namely amyloid plaques, neurofibrillary tangles, and AD-related cognitive impairment, has been verified. The abnormal cholinergic system regulates and promotes changes in amyloid precursor protein (APP) metabolism and tau phosphorylation, leading to neurotoxicity, neuroinflammation and neuronal death [37].

### 3.1. Amyloid Protein

Accumulation and deposition of amyloid beta and tau are key neuropathological features of neurodegenerative diseases such as AD. Interactions between oxidative stress, mitochondrial dysfunction, and misfolded tau and amyloid beta induced Ca^2+^ homeostasis damage play a crucial role in progressive neuron loss in specific brain regions [38]. Pathological features of AD also include neurofibrillary tangles (NTF) formation, synaptic dysfunction, and neuron loss [39,40]. P-tau is the major component of neuro-fibrous tangles (NFTs), and the accumulation of intracellular abnormal p-tau can lead to chronic traumatic encephalopathy (CTE) [41]. Hyperexcitability at the neuronal and network levels is usually associated with elevated Aβ levels [42]. Aβ can induce mitochondrial toxicity in the brain, resulting in cognitive dysfunction [5]. Abnormal accumulation of neurotoxic Aβ peptide is an early event of AD [43]. Abnormal Aβ is associated with deleterious changes in central cholinergic abnormality during the early stages of AD [44]. After injection of Aβ42 into the brain of male rats, the level of Aβ42 was significantly increased and the cholinergic function was decreased (the activities of acetylcholine and cholinesterase were decreased, and the activities of cholinesterase were increased), with changes of mitochondrial function, integrity, bioenergy and apoptosis. Aβ42 significantly reduced the expression level of heme oxygenase-1 in various brain regions of rats [45]. Aβand α-synuclein are two key proteins found in lesions associated with age-related neurodegenerative disease including AD [46]. The localization of Aβ plaques, particularly in regions rich in cholinergic neurons, led to the discovery that Aβ binds to a7 nAChRs with high affinity [47]. Amyloid precursor protein mediated disruption of the endocytosis pathway can induce axonal dysfunction and neurodegeneration. In the early stages of AD, the endosomal/lysosomal pathway is disrupted. Full-length APP and its β-C-terminal fragment lead to nutritional deficiency of BFCNs by increasing activation of Rab5, which leads to early endosomal enlargement and disruption of retrograde nerve growth factor (NGF) signaling and axon transport [48]. In addition, Aβ can significantly reduce spatial working memory and increase cholinergic dysfunction, decrease ChAT and ACh activity, and increase AChE activity in the hippocampus, prefrontal cortex, and amygdala; the degree of apoptosis is significantly increased in selected mouse brain regions. Moreover, Aβ significantly reduced mitochondrial function, integrity, and bioenergetics in all brain regions of mice [49]. The properties of mitochondrial dysfunction (MMP and RCR) were significantly associated with memory formation loss, Aβ levels and cholinergic dysfunction in these animals [4]. The apolipoprotein-ε4 (APOE4) allele is the only confirmed genetic risk factor for sporadic AD. Many reports have shown that amyloid-β interacts with butyrylcholinesterase and APOE. This interaction leads to the formation of soluble hyperactive acetylcholine hydrolytic complexes called BAβACs to modulate synaptic and extracellular ACh signaling as needed, with formation of BAβACs as allosteric regulators of cholinergic signal transduction. In summary, at least one intrinsic physiological function of Aβ is allosteric regulation of the intrinsic catalytic efficiency of cholinesterase, thus regulating synaptic and extra synaptic cholinergic signal transduction. High APOE may pathologically alter the biodynamics of Aβ function [50].

### 3.2. Neuroinflammation and Cholinergic System Abnormalities

Cholinergic atrophy and cognitive decline are accelerated in age-related neurodegenerative diseases such as AD [31]. Glutamate and cholinergic dysfunction are known features of AD [51]. Glutamate (Glu) and acetylcholine (ACh) are excitatory neurotransmitters that act through ionophilic receptors (iR) and metabolic receptors (mR). Neurotransmitters and their signaling are impaired in patients with AD [52]. AD is associated with cognitive dysfunctions such as memory loss caused by progressive deficits in cholinergic and glutamate signaling in the central nervous system [53]. Cognitive deficits are associated with the degeneration of cholinergic neurons [54], and the lack of the cholinergic system is believed to play a crucial role in cognitive impairment [55]. AD is involved in decompensation involving the structural and functional loss of cholinergic neurons, accelerating the neurodegenerative process [56]. ACh deficiency in AD patients leads to progressive and significant loss of cognitive and behavioral functions [57]. Damage to the cholinergic system may lead to increased cerebellar default mode network connectivity in AD [58].

#### 3.2.1. Acetylcholine Receptor and Signal Changing

The nicotinic acetylcholine receptor (nAChR) family is a prototypical member of the pentamer ligand gated ion channel, widely distributed in the central and peripheral nervous system, and its members are targets of genetic and acquired neurological disorders. In the central nervous system, nAChR is involved in the pathological mechanism of neurodegenerative diseases such as AD [59]. Through transdermal administration of the nAChR antagonist methylamine, functional magnetic resonance imaging showed that methylamine reduced nAChR tension in task-induced default mode network (DMN) inactivation, suggesting that continuous nAChR activation contributes to regulating DMN activity in healthy individuals. nAChR agonists modulate DMN by enhancing task-induced DMN deactivation. Low nAChR pitch has a causal role in DMN dysregulation seen in conditions such as mild cognitive impairment and AD [60]. Endogenous astrocytic α b-crystallin (Cryab) represses Aβ aggregation through regulation of nAChR activation and PI3K/Akt signal transduction in α7 neurons [61].

Disruption of cholinergic signals through muscarinic receptors has been associated with various pathologies including AD [62]. M1/M4 muscarinic receptors are potential targets of AD [63]. In a rodent model, activation of mGlu5 and M1 muscarinic acetylcholine receptor (mAChR) positive allogenic modulator (PAMs) induces a long-term depression (mLTD) in the mouse prefrontal cortex (PFC). Increased GABAa-dependent inhibitory tension in PFC pyramidal neurons leads to cortical disruption and schizophrenia [64]. GABA works in a non-synaptic manner to maintain immobilization of nerve stem/progenitor cells in the spinal cord. Pharmacological stimulation of cholinergic receptors and interference with GABAergic signals can promote functional recovery after spinal cord injury [65].

Changes in the ubiquitin-proteasome system (UPS) play a role in the early synaptic failure associated with the onset of AD. In cellular and animal models of AD, NGF-dependent changes in ubiquitin-homeostasis trigger early cholinergic degradation. In an in vitro model of a septal hippocampal neuron rich in cholinergic energy, it is found that damage to the NGF/TrkA signaling pathway triggers a “death” degradation process that occurs before cell death, and NGF tightly controls excitatory neurotransmission in vitro through ubiquitination mediated pathways, accompanied by loss of specific vesicle transporters [13]. The endocannabinoid system regulates emotional learning and memory through the CB1 receptor and has been found to be unregulated in AD. AD is characterized by progressive memory decline associated with selective impairment of cholinergic neurotransmission [66]. Cholinergic energy deficiency results in excessive depolarization of neuronal cells, leading to an excitatory toxic cascade induced by glutamate zinc. Intracellular zinc overload hits several neuronal targets, leading to energy balance breakdown and impaired function and structural damage of cholinergic neurons [67].

#### 3.2.2. Scopolamine Pathogenesis

Scopolamine is a muscarinic cholinergic antagonist that induces AD-like pathology in vivo and in vitro by altering the cholinergic system [9] and has therefore been used in numerous studies to understand, identify and characterize therapeutic targets for AD. Scopolamine acted on the brain region and reduced the protein expressions of phosphorylated Akt, ERK1/2 and CREB in the hippocampus, resulting in impaired memory and learning ability and significantly reduced hippocampal cholinergic system reactivity and neurogenesis [68]. Stress exposure enhances the amnesia effect of scopolamine: memory loss is induced by up-regulation of miR-1 and miR-10 in PFC and hippocampus, down-regulation of miR-1 and miR-26 in amygdala, and decreased protein expression. This suggests that cortical limbic signaling pathways may have a key function in the relationship between stress and AD [30]. Cortical auditory evoked potentials can be used as early biomarkers of cognitive impairment. The identification of neurophysiological biomarkers of mild cognitive impairment (MCI) will contribute to the detection and tracking of the progression of cognitive impairment [69]. Scopolamine could promote the production of LPO and reactive oxygen species (ROS), and scopolamine pretreatment increased apoptosis through oxidative stress-mediated activation of Bax and caspase-3, and mitochondrial dysfunction, thus inducing cytotoxicity. Scopolamine also down-regulated the protein levels of antioxidant enzymes superoxide dismutase, glutathione peroxidase, catalase, anti-apoptotic protein Bcl-2, and antioxidant proteins nuclear factor-like 2 (Nrf2) and HO-1. In addition, scopolamine inhibits the secretion of neurotrophic NGF and the growth of neuroblastoma cells, destroys synaptic integrity, mediates c-Jun N-terminal kinase (JNK) activation, and finally leads to synaptic dysfunction, neuroinflammation, and neurodegeneration [70,71]. Moreover, a scopolamine analogue H-89, as an inhibitor of protein kinase, can significantly reduce spatial learning ability, such as H-89-induced spatial learning disorder in male rats [72].

#### 3.2.3. Inflammation Pathogenesis

Impairment of forebrain cholinergic neurons regulating innate immune responses and inflammation leads to cholinergic dysfunction-related diseases including AD [73]. The basal forebrain contains the largest number of cholinergic neurons in the forebrain. BFCNs are involved in many cognitive functions, including attention, learning and memory, and are particularly vulnerable in AD [74]. The main outcome of the pathogenesis of AD is the destruction of cholinergic pathways in the cerebral cortex and basal forebrain [34]. Neuronal dysfunction in the denervated cortical region mediates degeneration of the basal forebrain cholinergic nucleus, leading to cognitive decline [75]. The loss of BFCNs can lead to impaired cholinergic neurotransmission, abnormal synaptic function and changes in structural lipid metabolism [76]. Cholinergic circuits project from the basal forebrain centers to the cerebral cortex and hippocampus [36]. In Down’s syndrome (DS) or trisomy 21, and AD-induced diseases, the BFCN projection system shows a degraded feature [77]. Sensorimotor networks are formed between different layers of the somatosensory cortex (SC), such as layer 5, and regions such as the NBM. Dysfunction of NBM, which is rich in cholinergic fibers, leads to reduced ACh release in SC and AD-like memory impairment. Electrical stimulation of NBM or irbotulin-induced NBM damage in rats has been found to lead to ADd-like neuropathic memory loss [78]. Survival, connectivity, and function of BFCN depend on NGF, which is retrograde from synthetic sites in the cortex and hippocampus. The form of NGF found in the human brain is proNGF. BFCNs are particularly susceptible to AD because they rely on retrograde nutritional support for proNGF signaling and transport [15]. Lack of NGF in the basal forebrain leads to degeneration of cholinergic neurons in the basal forebrain, which could lead to memory decline [7]. The NBM is the main source of cortical cholinergic innervation [79]. Degeneration of NBM cholinergic neurons and LC-NE neurons in the basal forebrain is an important cause of AD [11]. In the progression of AD, it is very easy to form Tau pathology and neurofibrillary tangles (NFT). Dysregulation of neurotrophic and neurotransmitter signal transduction is an early pathogenic mechanism, which is related to NFT formation in fragile BNM neurons and cognitive decline in AD [80].

Neuroinflammation is important in the onset and progression of neurodegenerative diseases such as AD. Liposaccharide (LPS) levels are higher in the brains of Alzheimer’s patients and are associated with neuroinflammation, which is controlled by neurocholinergic signaling, and cognitive decline [81,82]. Endogenous antibodies to signal molecules and receptors are associated with AD [83]. The hippocampus is severely affected in diseases with neuroinflammatory components such as AD [14]. Significant memory loss and cognitive decline are associated with neuronal death and gray matter loss, especially in the frontal cortex and hippocampus [84]. The destruction of neuronal homeostasis determines irreversible degeneration and neuronal apoptosis. This is also true in the case of altered NGF in sporadic AD, an age-related pathology characterized by cholinergic loss, amyloid plaques, and neurofibrillary tangles [85].

#### 3.2.4. Ion Instability Pathogenesis

Homeostasis of transition metal ions is believed to be related to the pathogenesis of AD [86]. Zinc plays an crucial role in neuronal signaling and neurotransmission, and the metal’s accumulation in the brain has been linked to neurological disorders such as AD. The AChE activity of Zn-treated cells is significantly increased. Zn treatment significantly reduced the level of glutathione and the activities of superoxide dismutase and catalase [87]. Iron balance disorder of iron dyshomeostasis is one of the main causes of neuronal death in AD [88]. Homeostasis of intracellular calcium levels is critical for maintaining cell structure and function. Homeostasis of intracellular Ca^2+^ levels is critical for maintaining cell structure and function. Manganese is a metal that is essential for basic physiological functions in the human body, but overexposure causes it to accumulate in multiple organs, especially the brain. Manganese builds up in the brain causing manganese poisoning, a form of Parkinson’s disease. In addition, manganese is a risk factor for several neurodegenerative diseases, including AD. Ca^2+^ regulation affects neurodegenerative processes and calcium signal interference may play a role in Mn neurotoxicity. The mechanisms of neurodegeneration induced by manganese include oxidative stress, free radical production and apoptosis [89]. Astrocytes play a critical role in brain homeostasis, and dysfunction of astrocyte Ca^2+^ physiology may result in nerves responding to neurotransmitters with Ca^2+^ elevation and releasing neurotransmitters that activate neuronal receptors and cause degenerative diseases [90]. The combination of low metabolism and autophagy deficiency may be a risk factor for AD. Changes in astrocytes’ cytoskeleton and form is an obvious characteristic, while oxidative stress defense and changes of cholesterol metabolism and gene transcription process are not so obvious.The influence of astrocyte inhibition changes can lead to local loop imbalance, resulting in larger more profound impact on the function of the neural network [21].

### 3.3. Other Factors

AD remains a mystery to researchers and clinicians. The onset of AD is insidious, progressive, and multifactorial. In addition to aging, a variety of other factors in life—mainly environment, lifestyle, chronic stress, multiple microbial infections and neuroendocrine function—can affect the immune system [91]. However, other pathophysiological features of AD have emerged, including neuroinflammation and dysregulation of the kynurenine pathway (KP) [92]. The importance of cerebrovascular dysfunction in the etiology and development of AD has been increasingly recognized [56]. Endothelial dysfunction plays an important role in the pathogenesis of AD. Patients with AD show a decrease in circulating endothelial progenitor cells (EPCs) that repair and maintain endothelial function [93]. Cognitive decline and memory impairment caused by oxidative brain injury are important pathological features of AD [70].

#### 3.3.1. Other Brain Damaging Cofactors

Clinical and experimental studies of aging have found many changes in the brain, such as reduced neurogenesis, increased synaptic defects, metabolic stress and inflammation associated with cognitive decline and neurobehavioral deficits. Although aging is not a disease, it is an important risk factor for deterioration of function, mood disorders, disease exaggeration, dementia and general disease susceptibility. In addition, life events related to mental stress and trauma can also lead to accelerated age-related diseases including AD [94]. Individuals with post-traumatic stress disorder (PTSD) are also a risk of developing various forms of dementia [95].

#### 3.3.2. Diabetes Related

Insulin resistance induced by a westernized diet is a risk factor for the development of AD, and LPS co-exists with Aβ1-42 in these patients [96]. Obesity has long been associated with AD, although the exact mechanism by which it affects cognition remains elusive and is the subject of current research [97]. In recent studies, interference with insulin receptor desensitization and downstream effects of insulin receptor signaling have been observed in the brains of patients with AD [98]. Changes in intestinal microbiota can lead to brain dysfunction in various neurological diseases, including AD [92]. Retinoids are vitamin A derivatives that interact with retinoid X receptors (RXRs) and retinoid receptors (RARs). Retinoids are mainly involved in neural patterning, differentiation, and axon growth. Impairment of retinoic acid signaling can lead to neurodegeneration and progression of AD [99].

#### 3.3.3. Pregnancy Related

Smoking during pregnancy has been linked to neurocognitive deficits. It has been suggested that dopamine (DA) neurons in the ventral tegmental region of the mesocortex limbic pathway play an important role in the pathological mechanisms induced by nicotine. Microarray analysis revealed that some of these DA genes were involved in the pathway of neurological diseases such as AD. Most of the significantly up-regulated/down-regulated genes found in DA neurons are related to G-protein-coupled protein receptor signaling and development [100]. Women with epilepsy often go into menopause early. Decreased estrogen levels are associated with increased neurodegenerative processes and cognitive decline. Estradiol (E2) enables the neurotransmitter genome structure to resist the damaging effects of epileptic persistence (SE). SE affects all major functional pathways, including apoptosis, AD, cell cycle, depression, synaptic vesicular cycle, glutamate, GABA, cholinergic, dopaminergic and serotonin neurotransmission [101].

## 4. Relevant Treatment of Alzheimer’s Disease

AD is a neurodegenerative disease recognized as the world’s most common type of dementia and the second leading cause of death. However, the specific pathogenesis of AD is still unclear. At present, several mainstream hypotheses have been recognized—the cholinergic hypothesis, amyloid β hypothesis, tau protein hypothesis and neuroinflammation hypothesis. At present, effective drugs and treatments for AD are very limited, so finding new effective drugs and promising treatments is a hot topic around the world. In this section, we will focus on the latest research in relation to several hypotheses, with a focus on the cholinergic hypothesis, providing a theoretical basis for clinical treatment and scientific research experiments.

### 4.1. Therapy Targeting the Cholinergic System

Cholinergic neurons are widely distributed across brain regions, which is significant in cognitive function. Normal cholinergic signal transduction related to learning and memory depends on ACh [11]. Basic forebrain cholinergic neurons (BFCNs) play an important role in learning, memory, and cognitive function [12]. Their survival and differentiation can be regulated by nerve growth factor (NGF). Cholinergic innervation is important in cortical and hippocampal areas involved in memory and learning processes. [13] When problems occur in the cholinergic system, the number of cholinergic neurons decreases. Impaired acetylcholine transferase activity and impaired ACh receptors are associated with a range of memory and cognitive impairments.

#### 4.1.1. AChEI

**Figure 2:**
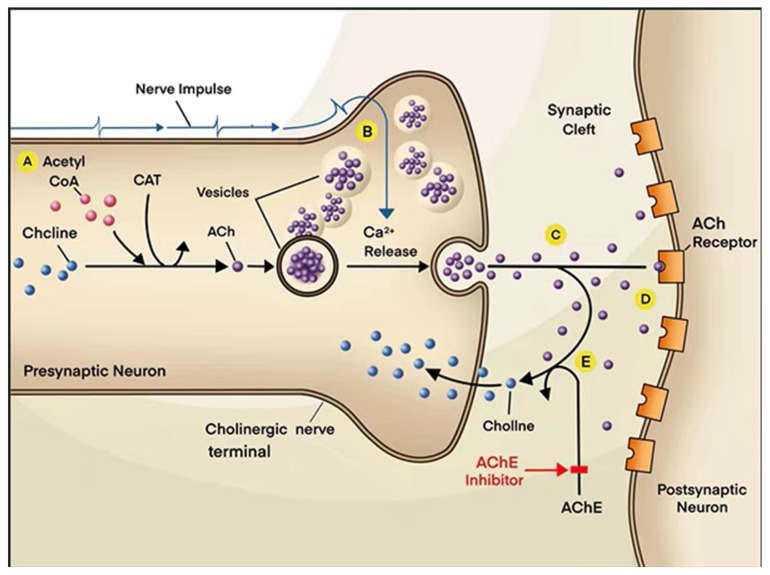
Schematic diagram of AChEI. ACh released from the presynaptic membrane into the synaptic cleft is normally rapidly hydrolyzed by AChE into choline. Choline is re-ingested by the presynaptic neuron to synthesize new ACh. People with AD have deficiency of ACh, and the levels of ACh in their brains are much lower than in normal people. AChEIs can inhibit the activity of AChE and thus block the hydrolysis of ACh, which can increase the concentration of ACh in the synaptic clefts and prolong the duration of ACh action in order to alleviate the cognitive impairment of AD patients. A, B, C, D, E refer to step A, B, C, D, E, which are the processes by which ACh is synthesized and metabolized in the body.

People with AD could develop severe ACh deficiency. At present, there are many drug therapies targeting AChE, which is the most common therapeutic target globally. AChE and butyrylcholinesterase (BChE) can potentially be used to treat AD. Green tea polyphenols as BchE and acetylcholinesterase inhibitors (AChEIs) can be used for the treatment of AD—the results of molecular docking showed that polyphenols exhibit interaction and inhibition by binding to AChE and BchE. Inhibition of AChE and BchE can prolong the time of cholinergic neurotransmission, which increases the cholinergic signaling. However, the AChE molecules still remain in the synaptic cleft (Figure 2). In view of these findings, cholinesterase inhibitors are recommended as therapeutic agents for the treatment of AD [56]. AChEIs are currently the only clinical drugs approved for the therapy of patients with AD [22]. AChEIs can relieve cognitive dysfunction in AD patients through inhibiting the degradation of Ach (Figure 2). The first generation of AChEIs was tacrine, which has been largely discontinued (Table 1). The most widely used AChEIs are the second generation of drugs including donepezil, rivastigmine and galantamine (Table 1) [21].

**Figure 3:**
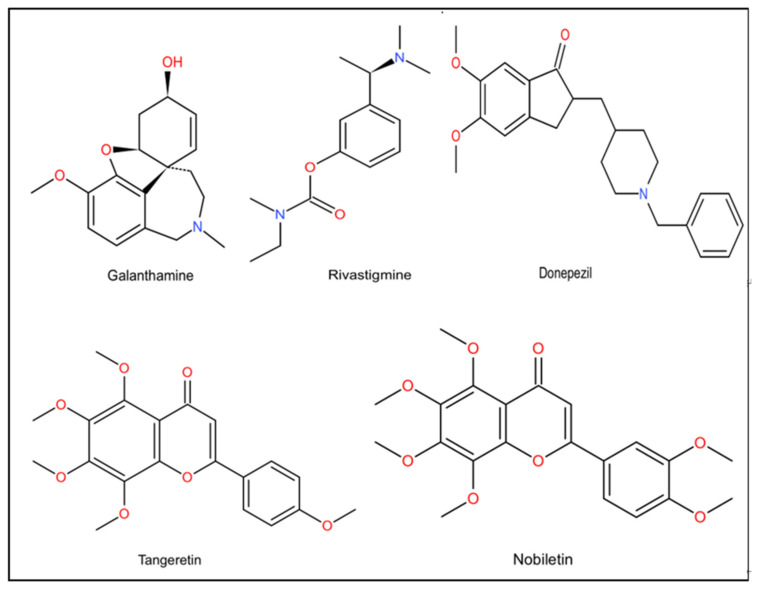
Some drugs related with the treatment of AD.

Donepezil is a clinically approved acetylcholinesterase inhibitor (AChEl) for the promotion of cognitive function in AD, and has been approved as a first-line drug for symptomatic therapy of AD (Table 1, Figure 3). The protein levels of PINK 1, NFASC, MYLK2 and NRAS in the hippocampus could be increased by donepezil. Donepezil can work through multiple mechanisms, which play an important role in protecting the neurons and nervous system. In particular, PINK 1 is associated with mitochondrial autophagy and cellular protective mitochondrial dysfunction, which may be significant in the pathogenesis of AD [102]. Compounds derived from alkaloids of plant phytochemicals could be used to treat AD, such as galantamine and carboplatin, to restore the decline of the cholinergic system [103]. Amyloid precursor protein (APP) and AChE are multifaceted proteins with a wide range of important functions, both of which are closely related to the pathogenesis of AD. APP is a precursor of Aβ peptide associated with AD, while AChE is related to its pathogenesis by reducing cholinergic deficiency or aggravating Aβ fibrillogenesis and toxicity [104]. Rivastigmine is currently approved as a drug for the symptomatic treatment of mild to moderate AD (Table 1, Figure 3). β-Amyloid (Aβ) is produced from its APP by endoproteolysis of β secretase (or BACE1) and Y-secretase. Alternative APP cleavage via a secretase (a membrane-bound family of metalloproteinases—Adamalysine) eliminates toxic Aβ production and produces neuroprotective and neurotrophic secretory sAPPa fragments. In addition to anticholinesterase activity, rivastigmine also guides APP treatment away from BACE1 and toward A secretase [105]. Patients treated with rivastigmine and galantamine had better daily living experiences and overall functioning than those treated with donepezil (Table 1, Figure 3) [21].

In addition to the four FDA approved AChEIs mentioned above for the treatment of AD [109], there are other promising drugs that have recently been used for clinical treatment and are under investigation. Huperzine A (HupA), a traditional Chinese medicine, is extracted from Huperzia Serrata shrimp, has been used to treat fever and inflammation in China. It can act as an AChEI to treat AD and other forms of dementia. It provides protection against excitatory toxicity and death of neurons, as well as increased GABA-energy transmission associated with anticonvulsant activity [110]. Intracerebral neurostructural and functional changes induced by streptozotocin were similar to those in patients with sporadic AD, manifested by decreased ACh level and increased AChE activity. Valproic acid (VPA), a histone deacetylase inhibitor (HDACi), restored ACh levels and normalized AChE activity in ICV-STZ injected mice [98]. Currently, compounds derived from isopropazine have been identified as effective cholinesterase inhibitors. They further showed anti-Aβ aggregation activity, anti-cholinesterase and antioxidant potential in vitro, showing a certain medical prospect for the treatment of AD [111].

According to the recent data, AChEIs have a moderate efficacy in the treatment of AD dementia, but the effect is not sustained. Most clinically used cholinesterase inhibitors (ChEl) have achieved limited clinical results [112]. These drugs can reduce or temporarily slow the symptoms of AD, but they do not prevent brain damage [109]. In 2012, the French Pharmacoeconomic Committee downgraded the rating of medical benefits provided by AChEIs in AD from “major” to “low” [21]. In 2018, the French ministry of health decided to delete several items from the available drug reimbursement list of drugs to treat AD symptoms (namely donepezi, rivastigmine, galantamine, memantine) due to the significant side effects, lack of medical benefits and risk [113]. However, since the causes of AD still remain unclear, AChEIs are still the main drugs in the clinical treatment of AD.

#### 4.1.2. Improvement of Deficiency in ACh

Weakness of the cholinergic system is thought to play a crucial role in cognitive impairment in dementia [55]. Therefore, reducing cholinergic deficiency is an important treatment for AD. A deficiency of the cholinergic system implies a deficiency of acetylcholine. ACh is known to drive the brain’s state of attention and arousal, which is lacking in diseases such as AD. Vascular signals can be shown by brain imaging techniques to map changes in neuronal activity, relying on coupling between electrophysiology (local field potentials, LFPs) and hemodynamics (CBF), a phenomenon known as “neurovascular coupling” (NVC). Neurovascular coupling is defined as the close relationship between activated neurons and hemodynamic responses, which is the basis of brain function imaging based on hemodynamics. ACh is not only an effective modulator, but also a necessary condition for the full expression of sensation-induced neurovascular coupling responses. Induced changes in neuronal activity can be assessed by ACh through altering the fidelity of hemodynamic signals [20].

Currently, chemicals derived from natural plants to treat AD are considered a promising field of medicine. Herbal remedies are now considered as complementary and alternative interventions for AD [114]. Nobiletin and tangeretin are important citrus flavonoids from Citrus L. Pericarp and other parts of the genus and have been shown to protect the neurons and nervous system in in vitro and in vivo studies (Figure 3). Besides antioxidant and anti-inflammatory effects, nobiletin and tangeretin have been indicated to abate the lack of choline, reduce abnormal accumulation of amyloid peptide, reverse the decrease of N-methyl-aspartate (NMDA) receptor, improve ischemic injury, inhibit Tau protein phosphorylation, enhance the level of neuropeptides, adjust some signaling cascades, and prevent toxicity of 1-methyl-4-phenylpyridine (MPP (+) and 1-methyl-4-phenyl-1, 2, 3, 6 tetrahydrogens (MPTP) (Figure 3) [115]. The seed of Litchi chinensis fraction (SLF), a well-known traditional Chinese medicine, has recently been indicated to promote cognition through inhibiting neuronal apoptosis in rats. SLF has been shown to play a neuroprotective role in AD rats through the Akt-3 β signaling pathway, thus proving that SLF is a potential drug for the treatment of AD [116]. β-amyrin, a component of the surface wax of tomato fruits and dandelion coffee, has previously been reported to improve memory impairment caused by cholinergic dysfunction. β-amyrin ameliorates object recognition memory deficits in an Aβ-injected mouse model of AD. Moreover, Aβ-induced neurogenesis disorders were ameliorated by amyrin therapy [1]. Taken together, these phytochemicals derived from natural plants could represent novel drug candidates for the treatment and prevention of AD.

At the same time, some new drugs and treatment opportunities have also aroused the interest of researchers. A novel T-type Ca^2+^ channel enhancer improves cognition and inhibits amyloid accumulation in a AD mouse model. This type of channel enhancer increases the level of ACh and neurogenesis in the hippocampus [117]. Maternal choline supplementation (MCS) can supplement the reduced ACh levels in the brain of AD mice. This therapeutic intervention has previously been indicated to reduce spatial, cognitive and attentional dysfunction in Ts65Dn mouse models and to have a protective effect on basal forebrain cholinergic neuron survival. MCS is a potential treatment for neuronal signaling dysregulation in the trisomic mouse brain through long-term reprogramming of key transcripts, which have the potential to translate into AD progesterone (P4) and 17p estradiol (E2) [118]. The neuroprotective effects of P4 and E2 were highly correlated with the improvement of cholinergic deficiency, inhibition of apoptotic signals and astrocytomiosis, and up-regulation of 5-HT2A expression. Therefore, HRT can be considered as an effective therapy for treating patients with cognitive impairment and neurodegenerative diseases [119]. Translational ribosomal affinity purification (TRAP) was coupled with RNA sequencing (Trap-SEQ) to identify actively translated mRNAs in forebrain cholinergic neurons in AD’s TgCRND8 mouse model. Bioinformatics analysis revealed downregulation of 67 of 71 known cholinergic related transcriptions compared with those in TgCRND8 mice. Trap-seq identified differential gene expression changes in TgCRND8 mouse cholinergic neurons compared with wild-type modulator, providing a new candidate pathway for the therapeutic development of AD [120]. Although restoration of cholinergic system function is not expected to fully relieve cognitive impairment in AD, key aspects of cognition, including prodromal attention deficit, are likely to offer openings for corrective measures [121].

#### 4.1.3. Protection of BFCNs and Regulation of NGFs and BDNF

Basic forebrain cholinergic neurons (BFCNs) play an important role in learning, memory, and cognitive function. Dysfunction or degeneration of BFCNs may be associated with neurodegenerative diseases, such as AD. The production of functional BFCNs may be significant in the study of cell-based therapy and pathogenesis associated with defects in learning and memory [12]. The survival and differentiation of basal forebrain cholinergic neurons (BFCNs) depend on nerve growth factor (NGF) [13]. Patients with AD have degeneration of basal forebrain cholinergic neurons and degeneration of cholinergic neurons. NGF enhances the survival and function of cholinergic cells, which improves memory [122]. Neurotrophic molecules contribute to maintaining the integrity and function of cholinergic neurons during development and adulthood. Neurotrophic factors such as NGF and brain-derived neurotrophic factor (BDNF) have gradually become dysregulated in the case of AD [36].

Citicoline is a compound from natural plants and an FDA-approved dietary supplement.It is a forebrain agent that has currently been shown to be therapeutic in improving ischemic stroke, traumatic brain injury, Parkinson’s disease, AD, and cerebrovascular disease. Phosphocholine is a neuroprotective agent, nerve recovery and nerve regeneration agent [123]. Donepezil seems to significantly up-regulate the expression level of BDNF and phosphorylation level of TrkB by regulating the cholinergic system and inhibiting the BDNF/TrkB dependent signaling pathway, thus protecting glial cells from aging, inhibiting neuronal damage, and improving learning and cognitive disorders [124]. Early cholinergic degradation is triggered by NGF-dependent changes in ubiquitin-homeostasis in AD model animals. In an vitro model of a septal hippocampal neuron rich in cholinergic energy, we found that damage to the NGF/TrkA signaling pathway triggers the “death” degradation process that occurs before cell death. NGF tightly controls excitatory neurotransmission in vitro through ubiquitination mediated pathways, accompanied by loss of specific vesicle transporters. The important role of UPS in neurogrowth factor/TrkA signaling dysregulation in cholinergic synaptic properties provides a new therapeutic target for AD, which is a new direction for the treatment of AD [13] and a potential novel way for neural stem cells (NSC) to be used for the treatment of AD. However, due to the low rate of neural differentiation, the therapeutic effect is moderate. Neurotrophin-3 (NT-3) can promote the proliferation and differentiation of BM-NSC neurons into cholinergic neurons and increase the level of ACh in the supernatant. NT-3 has the same therapeutic potential as subventricular NSCs [125]. Purmorphamine has been developed to induce human pluripotent stem cells to differentiate basal forebrain cholinergic neurons in response to dysfunction and degeneration of BFCNs in AD [12]. This suggests that the induction of directed differentiation of pluripotent stem cells and the addition of BFCNs is a feasible research direction, providing a potential application prospect for the study of disease treatment. AD can cause memory loss and cognitive deficits. One promising area is substances and compounds derived from natural plants that can provide broad neuroprotection against AD melatonin (MEL) and resveratrol (RES), which both enhance the cholinergic system and BDNF and CREB signaling pathways in the prefrontal cortex [126]. 7,3′,4′-trihydroxyisoflavone trihydroxyisoflavone (THIF) is a secondary metabolite derived from daidzein that regulates cholinergic and BDNF signaling to improve cognitive function [127]. Rh2 is a rare ginsenoside that can regulate cholinergic transmission, inhibit oxidative stress and activate the ERK-CREb-BDNF signaling pathway. Therefore, ginsenoside Rh2 may be a promising candidate compound for AD [125].

#### 4.1.4. AChR and Scopolamine

Disruption of cholinergic signals through muscarinic receptors has been associated with various pathologies, such as AD [62]. Muscarinic acetylcholine receptor (mAChR) contributes to the regulation of complex behaviors such as cognition, movement, and reward, and provides an ideal site for potential drug targets for several brain disorders [128]. TAK-071 is a muscarinic M1 receptor positive allosteric regulator, which has low synergism with ACh and can safely enhance the functional connections of neurons [129]. mAChR allosteric modulators have entered into the clinical development stage for use in AD and have potential effects on other brain diseases [128]. M1 muscarinic acetylcholine receptor (M1 mAChR) is a potential alternative therapeutic target. The M1 mAChR ligand promotes phosphorylation-dependent signal transduction of receptors, preventing cholinergic adverse reactions in addition to beneficial responses associated with AD therapy, such as learning, memory, and anti-anxiety behaviors [130]. Hippocampal cholinergic neurostimulating peptide (HCNP) is a kind of cholinergic agonist that acts through M1 mAChR [131], which could become a valuable candidate drug.

Nicotinic acetylcholine receptor (nAChR) family is a prototypical member of pentamer ligand gated ion channel, and its members are targets of genetic and acquired neurological disorders. Its distribution is wide in the central and peripheral nervous system. In the central nervous system, nAChR contributes to the pathological mechanisms of neurodegenerative diseases such as AD. The default mode network (DMN), a group of brain systems that are more active at rest and mediate task-independent thought processes [59], has been implicated in degenerative diseases. It has been found experimentally that low nAChR level in the condition of AD can lead to DMN dysfunction [60]. nAChR has also been considered as a potential molecular target for the treatment of cognitive dysfunction in AD because it is located in key regions of the brain for learning and memory [132].

Scopolamine is a muscodamine cholinergic antagonist, which can promote the production of LPO and ROS and aggravate the cognitive decline and memory impairment caused by oxidative brain injury. Meanwhile, it can also destroy the integrity of synapses and lead to synaptic dysfunction [70]. Scopolamine has been used in many research studies to explore and identify therapeutic targets for AD [71]. Rutin improves cognitive dysfunction in scopolamine-induced amnesia by enhancing antioxidant defense and cholinergic systems [133]. 7,3′,4′-trihydroxy isoflavones (THIF) can effectively improve the memory impairment induced by scopolamine in mice. Biochemical tests of the entire hippocampus of the mice showed that 7,3′,4′-trihydroxyisoflavones restored the cholinergic damage induced by scopolamine [127].

### 4.2. Relevant Treatment for Neuroinflammation

Neuroinflammation is significant in the onset and development of neurodegenerative diseases such as AD. Neuroinflammatory responses found in the brain are thought to be involved in the pathogenesis of AD [86,134]. Apelin-13 is a major neuropeptide that inhibits inflammation and plays a role in cognitive memory and neuron damage [81]. Retinoids are mainly related with neural patterning, differentiation and axon growth, and damage of retinoid signaling can lead to neurodegeneration and progression of AD. It also has been indicated to inhibit the expression of chemokines and pro-inflammatory cytokines in microglia and astrocytes that are activated in AD [99]. Galantamine reduced inflammatory responses not only in the BV-2 microglia cell line, but also in the HT-22 hippocampal neuron cell line, improving cognitive decline and neuroinflammation in endotoxin-induced neurodegenerative diseases. Galantamine may thus be a promising treatment [82]. Ellagic acid (EA) is a phenolic plant component extracted from grains and fruits, which has significant antioxidant effects and can effectively regulate endogenous molecular signals in the human body. EA treatment normalized the abnormal behaviors associated with sporadic AD (SAD) in rats, suggesting that EA can improve neuroprotective effect and cognitive behavior in SAD rats. Curcumin can treat AD due to its anti-inflammatory effect [135]. Curcumin is not only an effective PPARγ agonist, but also has a neuroprotective effect on ischemic brain injury. Curcumin also reduced microglia and astrocyte activation, as well as cytokine production and inhibition of the nuclear factor Kappa B (NF-κB) signaling pathway, indicating that the beneficial effects of curcumin on AD can be attributed to inhibition of neuroinflammation. PPARγ may be a potential target of curcumin, alleviating neuroinflammation and improving neuronal function in AD [134]. AD-35, a patented small-molecules compound derived from novel modification of the chemical structure of donepezil, could target metal-Aβ species and neuroinflammation. AD-35 modestly inhibited AChE and metal-induced Aβ aggregation in vitro and demonstrated deconstruction of Aβ depositions. Oral administration of AD-35 significantly attenuated injection-induced astrocyte activation, release of pro-inflammatory cytokines TNF-α and IL-1β, and memory deficits [86].

### 4.3. Reduction of Deposition of Amyloid Beta (Aβ) and Phosphorylation of Tau

Accumulation and deposition of β amyloid and tau proteins are key neuropathological features of neurodegenerative diseases such as AD. How to reduce the accumulation and deposition of amyloid beta and phosphorylated tau protein is one of the problems to be considered in the treatment of AD. Mice with AD treated with isoastilbin (IAB) had less time to escape from the water maze and more ability to search for hidden food in the Y maze, indicating that the abnormal behavior of mice was improved. Through further research, it was found that IAB decreased the deposition of amyloid beta (Aβ) and phosphorylated tau expression in mouse brain, and increased serum Aβ level [136].

Several treatments are also being used to reduce the accumulation and deposition of amyloid beta and phosphorylated tau proteins to treat AD. Acupuncture is a famous traditional Chinese medicine treatment. Clinical data indicates that electroacupuncture (EA) first enhances and then attenuates the signaling cascade triggered by collagen II-combined complete Freund’s adjuvant, leading to oxidation, nitrosylation, hypoxia, and angiogenesis. Ultimately, EA inhibits neurodegeneration by reducing amyloid beta peptide (Aβ) and phosphorylated tau (P-Tau), and also corrects neuronal dysfunction by increasing the cholinergic neurotransmitter ACh and its rate-limiting biosynthase choline acetyltransferase (ChAT) [137]. Currently, a novel T-type Ca^2+^ channel enhancer has been found to promote cognition and inhibit β-amyloid accumulation in a mouse model of AD. This type of channel enchancer stimulates ACh release and neurogenesis in the hippocampus, which can improve the cholinergic system [117].

Natural remedies are now gaining increasing acceptance by consumers all over the world. Global public health advocates daily consumption of fruits and vegetables to prevent cardiovascular disease and stroke. Polyphenols in fruits and vegetables have strong antioxidant and anti-inflammatory properties and have been shown to regulate tau hyperphosphorylation and amyloid beta accumulation in animal models of AD [138]. Medicinal plants and herbal remedies are now considered as complementary and alternative interventions for AD [114]. The aforementioned green tea polyphenols containing BchE and AChEIs can be used for prophylaxis. These findings indicate that natural remedies are a valuable source for AD prophylaxis.

### 4.4. Other Treatment for AD

Blood from young individuals is beneficial for age-related diseases. Recent research showed that young serum intravenously injected attenuated defects in learning and memory function, alleviated hippocampal Aβ plaque deposition, restored synaptic formation and synaptic plasticity, and repaired cholinergic circuits in the hippocampus. Moreover, it could induce several regulatory neuroprotective mechanisms in elderly AD model mice. Exogenous young serum can plays a therapeutic role in AD related cognitive impairment, promoting hippocampal cholinergic input and simultaneously activating other neuroprotective mechanisms [139]. Women with epilepsy often experience early menopause. Decreased estrogen levels are related with aggravated neurodegenerative processes and cognitive dysfunction. Estradiol (E2) enables genomic fabrics-related neurotransmission to reverse the harmful effects of status epilepticus (SE) [101]. This indicates that estrogen plays an importance in protecting the nervous system against neurodegenerative diseases. Smoking during pregnancy has been linked to neurocognitive deficits in babies. Dopamine (DA) neurons in the ventral tegmental region in mesocorticolimbic pathway are significant in nicotine-induced neurocognitive deficits. Microarray analysis revealed that gestation nicotine exposure could cause the alteration of some genes in DA neurons related to neurological disease pathways, such as AD [100]. For the health of the baby, women during pregnancy should avoid smoking and remain in a smoke-free environment as far as possible.

Although there are many promising drugs and treatments, AChEIs are the only drugs currently approved for the therapy of AD. Because AD is an extremely complex and irreversible neurodegenerative disease affected by both genetic and environmental factors, we still do not know much about it. Existing drugs often target only one cause of the disease and often do very little. There is an urgent need to research and develop clinical drugs related to AD; up to the present, clinical data on drugs are very limited, unrepresentative and unpersuasive. Moreover, current drugs can only relieve symptoms, not prevent or delay the disease. With the increasing number of patients with AD, the treatment of AD in the future will become an increasingly hot topic globally.

## 5. Conclusions

This review attempts to summarize the importance of cholinergic signaling in AD and briefly present the latest drugs and treatments for AD, in order to provide theoretical support for further research and treatment for AD. Based on the above, it is clear that the cholinergic signaling is significant in AD. Cholinergic neurons are distributed widely in brain regions and play a role in cognitive function. ACh can regulate the normal cholinergic signal transduction associated with learning and memory. Patients with AD often manifest deficiency in ACh and damage to cholinergic signal transduction. Therefore, cholinergic signaling is a critical target for treatment of AD. Although there are currently some drugs available to treat AD, further research is needed to develop more effective therapeutic agents.

## Figures and Tables

**Table 1:** Summary the AChEI drugs approved by FDA.

Classification	Name	Property	Reported Correlation with AD	Adverse Reaction	References
First generation	Tacrine	Reversibility, Lipid solubility	Inhibits AChE in plasma and tissues. Promotes ACh release through M1 receptor.	Hepatotoxicity	[106]
Second generation	Donepezil	Reversibility, High selectivity	The only inhibitor that can act on both the peripheral and central catalytic sites of AChE. Increases the concentration of AChE by reversibly inhibiting the ACh hydrolysis induced by AChE. Increases the protein levels of PINK 1, NFASC, MYLK2 and NRAS in the hippocampus.	Nausea, Vomiting Diarrhea Fatigue	[102]
Second generation	Rivastigmine	Reversibility	Guides APP treatment away from BACE1 and toward A secretase. Increases the concentration of AChE by reversibly inhibiting the ACh hydrolysis induced by AChE.	Dizziness Vertigo Upper respiratory tract infection	[21,105]
Second generation	Galantamine	Reversibility	Allosteric activation effect on nicotinic ACh receptors. Activates MARK, PI3K and other cell signal transduction pathways to play an anti-inflammatory effect. Promotes the release of neurotransmitters associated with glutamate, norepinephrine and memory and mood. Protects nerves against oxidative damage caused by hydrogen peroxide.	Salivation Bradycardia Dizziness Abdominal pain	[107,108]

## Data Availability

Not applicable.

## Sample Availability

Samples of the compounds are available from the authors.
